# EHMTI-0215. The impact of post-herpetic neuralgia on patients with chronic primary headaches

**DOI:** 10.1186/1129-2377-15-S1-D28

**Published:** 2014-09-18

**Authors:** A Avetisyan, AS Harutyunyan, HR Vekilyan, AH Karapetyan, EM Gevorgyan, HM Manvelyan

**Affiliations:** 1 Neurology, Yerevan State Medical University after M.Heratsi, Yerevan, Armenia; 2 Neurology, 2nd hospital, Yerevan, Armenia

## Introduction

Increasing number of patients with post-herpetic neuralgia (PHN) leads to clinical observation of the fact that it worsening the condition of patients with other chronic pain syndromes such as migraine and tension-type headache (TTH).

## Aims

The aim of this study was assessment of cephalgic pain severity and quality of life (QoL) in patients with primary headaches in comparison with those who additionally suffers of PHN.

## Methods

12 patients with PHN and comorbid migraine and TTH (3 and 9, respectively) and 14 patients with migraine and TTH only (4 and 10) as controls were assessed for headache pain severity by visual analog scale (VAS) and QoL by short form (SF-36) screening tools.

## Results

Patients with PHN had worse results of headache pain intensity and QoL vs patients of control group. Headache pain severity in PHN group was 7-8 vs 5-6 in control group, and QoL scoring was lower in PHN group vs control.

## Conclusion

PHN as the reason of central sensitization leads to further increase of the other chronic pain syndromes, such as primary headaches, elevate the cephalgic pain and decrease the QoL.

No conflict of interest.

